# Ezabenlimab (BI 754091), an anti-PD-1 antibody, in patients with advanced solid tumours

**DOI:** 10.1007/s00262-024-03654-0

**Published:** 2024-03-30

**Authors:** Manish R. Patel, Melissa Johnson, Ira Winer, Hendrik-Tobias Arkenau, Natalie Cook, Vanessa Samouëlian, Raid Aljumaily, Shigehisa Kitano, Christine Duffy, Miaomiao Ge, Mabrouk Elgadi, Lillian L. Siu

**Affiliations:** 1 Sarah Cannon Research Institute, 250 25th Ave N, Nashville, TN 37203 USA; 2 Florida Cancer Specialists, 600 N Cattlemen Rd, Suite #200, Sarasota, FL 34232 USA; 3 Tennessee Oncology, Nashville, TN USA; 4 Wayne State School of Medicine, Karmanos Cancer Institute, Detroit, MI USA; 5 Sarah Cannon Research Institute, Cancer Institute, University College London, London, UK; 6 The Christie NHS Foundation Trust and the University of Manchester, Manchester, UK; 7 Centre Hospitalier de L’Université de Montréal, Montreal, QC Canada; 8 Stephenson Cancer Center of the University of Oklahoma and Sarah Cannon Research Institute, Oklahoma City, OK USA; 9 Japanese Foundation for Cancer Research, Tokyo, Japan; 10 National Cancer Center Hospital, Tokyo, Japan; 11 Boehringer Ingelheim Pharmaceuticals Inc., Ridgefield, CT USA; 12 Princess Margaret Cancer Centre, University Health Network, Toronto, ON Canada

**Keywords:** Ezabenlimab, Advanced solid tumours, PD-1 inhibitor, Phase I

## Abstract

**Background:**

Ezabenlimab (BI 754091) is a humanised monoclonal antibody targeting programmed cell death protein-1. We report results from open-label, dose-escalation/expansion, Phase I trials that evaluated the safety, maximum tolerated dose (MTD), pharmacokinetics and antitumour activity of ezabenlimab at the recommended Phase II dose in patients with selected advanced solid tumours.

**Study design:**

Study 1381.1 (NCT02952248) was conducted in Canada, the United Kingdom and the United States. Study 1381.4 (NCT03433898) was conducted in Japan. Study 1381.3 (NCT03780725) was conducted in the Netherlands. The primary endpoints were: number of patients experiencing dose-limiting toxicities (DLTs) in the first cycle (dose escalation parts), number of patients with DLTs during the entire treatment period and objective response (dose expansion part of Study 1381.1).

**Results:**

Overall, 117 patients received ezabenlimab intravenously every 3 weeks (80 mg, n = 3; 240 mg, n = 111; 400 mg, n = 3). No DLTs were observed and the MTD was not reached. Fifty-eight patients (52.3%) had grade ≥ 3 adverse events, most commonly anaemia (10.8%) and fatigue (2.7%). In 111 assessed patients treated with ezabenlimab 240 mg, disease control rate was 56.8% and objective response rate was 16.2%. Three patients had complete response; at data cut-off (November 2021) one remained in response and was still receiving ongoing treatment (duration of response [DoR]: 906 days). Partial responses occurred across several tumour types; DoR ranged from 67 to 757 days.

**Conclusions:**

Ezabenlimab was well tolerated and associated with durable antitumour activity in multiple solid tumours, comparable to other immune checkpoint inhibitors in similar patient populations and treatment settings.

**Supplementary Information:**

The online version contains supplementary material available at 10.1007/s00262-024-03654-0.

## Introduction

Immune checkpoint inhibitors have revolutionised the treatment of cancer over the last decade. Monoclonal antibodies that target programmed cell death protein-1 (PD-1; e.g. nivolumab and pembrolizumab) and its ligand, PD-L1 (e.g. atezolizumab and durvalumab), have been shown to be promising anticancer strategies and thus are approved for the treatment of a wide variety of solid tumours haematological malignancies [1]. Although anti-PD-1/PD-L1 therapies have achieved great success in treating cancers, only a subset of patients show clinical response [2–6]. Furthermore, a large group of responders develop acquired resistance after initial response and require alternative therapeutic approaches.

Ezabenlimab (BI 754091) is a humanised PD-1-targeting monoclonal antibody [7]. Here, we report safety, pharmacokinetic and efficacy results in patients with advanced solid tumours who received ezabenlimab monotherapy in three open-label, Phase I studies (Studies 1381.1 and 1381.4, and one patient from Study 1381.3), with a focus on patients who received ezabenlimab monotherapy at the recommended Phase II dose (RP2D) of 240 mg given every 3 weeks (Q3W).

## Methods

### Study design and patients

This manuscript reports data from three open-label, Phase I studies assessing ezabenlimab monotherapy in patients with advanced solid tumours. The international, multi-cohort Study 1381.1 (NCT02952248) was conducted in Canada, the United Kingdom and the United States and comprised dose escalation and dose expansion parts. Study 1381.4 (NCT03433898) was conducted in Japan and assessed ezabenlimab as monotherapy and in combination with BI 754111, a lymphocyte activation gene 3 (LAG3) inhibitor. Additionally, one patient from imaging study 1381.3 (NCT03780725), which was conducted in the Netherlands, was included [8]. The studies were conducted in accordance with the Declaration of Helsinki and Good Clinical Practice Guidelines. All patients provided written informed consent.

Eligible patients were aged ≥ 18 years old, with an Eastern Cooperative Oncology Group performance status (ECOG PS) of 0 or 1, and adequate organ function. For the dose-escalation/dose-finding parts of Studies 1381.1 and 1381.4, patients had a histologically or cytologically confirmed diagnosis of advanced, unresectable or metastatic solid tumours, and had disease progression on, were intolerant to, or not eligible for, standard therapies, including immune checkpoint inhibitors (anti-PD-1, anti-PD-L1, or cytotoxic T-lymphocyte–associated protein 4 inhibitors). In the dose expansion part of Study 1381.1, patients were enrolled into one of four cohorts: Cohort 1, seven solid tumour types (non-small cell lung cancer, bladder cancer, melanoma, gastric cancer, ovarian cancer, triple-negative breast cancer and renal cell carcinoma); Cohort 2, tumours with high tumour mutational burden (TMB; ≥ 10 mutations/MB) excluding those with high microsatellite instability; Cohort 3, squamous cell cervical, anal and skin tumours that were refractory to standard therapies; and Cohort 4, recurrent human papilloma virus-positive or -negative vaginal or vulvar squamous cell carcinoma (VSCC) not amenable to surgery. All patients in the dose-expansion cohort of Study 1381.1 were required to have measurable lesions per Response Evaluation Criteria in Solid Tumours (RECIST) version 1.1, have at least one tumour lesion amenable to biopsy, and be willing to undergo a biopsy before first treatment and after 6 weeks of therapy. All patients had either progressed on conventional treatment, were not amenable to standard therapies, or there were no available therapies of proven efficacy.

Key exclusion criteria included: previous treatment with an anti-PD-1, anti-PD-L1, or cytotoxic T-lymphocyte–associated protein 4 inhibitor, requirement for chronic systemic steroid therapy, history of pneumonitis, and presence of untreated active brain metastases or active autoimmune disease or infections.

### Treatment

The dose escalation part of Study 1381.1 was guided by a Bayesian Logistic Regression Model (BLRM) with overdose control, with cohorts of three patients sequentially enrolled at ezabenlimab doses of 80, 240, and 400 mg (predetermined maximum administered dose) administered intravenously over 60 min on Day 1 of 21-day cycles.

In the dose expansion part of Study 1381.1, part 1 of Study 1381.4 and in the one patient included from Study 1381.3, ezabenlimab was administered at 240 mg Q3W (the RP2D established during dose escalation).

Treatment was continued for up to 1 year, or until disease progression or unacceptable toxicity. Treatment could also be continued beyond 1 year in patients with clinical benefit. Patients could remain on treatment at the investigator’s discretion following initial radiological progressive disease.

### Endpoints and assessments

In the dose-escalation/dose-finding parts of Studies 1381.1 and 1381.4, the primary endpoint was the number of patients experiencing dose limiting toxicities (DLTs) in the first cycle. Secondary endpoints included pharmacokinetic parameters, DLTs in all cycles and objective response rate (ORR). For the dose expansion of Study 1381.1, the primary endpoints were number of patients with DLTs during the entire treatment period and ORR. Secondary endpoints included progression-free survival and safety.

Safety was assessed by descriptive analysis of incidence and severity of adverse events (AEs; graded per Common Terminology Criteria for Adverse Events version 5.0). Investigators indicated whether an AE was a potentially immune-related event. Immune-related AEs are AEs associated with immunotherapy treatments that appear to be associated with the immune therapy’s mechanism of action. In general, treatment of low grade immune-related AEs was left up to the discretion of the investigator. The protocol provided guidelines for management of potentially life-threatening AEs (such as pneumonitis, colitis, encephalitis, diabetes, hepatitis, etc.) consistent with international guidelines. The Phase I trial statistical analysis plan did not include specific analysis for time to resolution of low-grade AEs.

Blood samples were collected at pre-defined timepoints during each study to evaluate the presence of anti-drug antibodies (ADAs) using a validated immunoassay in a tiered approach. In Study 1381.1, biomarker assessments were performed in both blood samples (for PD-1 receptor occupancy in peripheral blood mononuclear cells in the dose escalation phase and cytokine analysis in the dose expansion phase) and tumour biopsies (for immunohistochemical analysis of PD-L1, PD-1, LAG-3 and CD-8). Tumour assessment was performed at baseline, every two cycles during the first 6 months and every three cycles thereafter. Response was evaluated by the investigator per RECIST version 1.1 and iRECIST (modified RECIST version 1.1 for immune-based therapeutics).

Blood samples were also analysed to evaluate pharmacokinetics. Ezabenlimab plasma concentrations were evaluated using a validated immunoassay.

A population pharmacokinetic two-compartmental model was developed using the nonlinear mixed effects modelling approach. Data from 66 patients from BI Studies 1381.1 and 1381.2 (a Phase I dose-finding study of BI 754111 in combination with ezabenlimab) were used in the model. The predicted pharmacokinetic profile at steady state for ezabenlimab 240 mg Q3W was compared with that of pembrolizumab 200 mg Q3W.

### Statistical analysis

The dose escalation part of Study 1381.1 was guided by a BLRM with overdose control. The estimated probability of a DLT at each dose level from the model was summarised using the following intervals: under dosing (0.00–0.16), targeted toxicity (0.16–0.33) and over toxicity (0.33–1.00). The BLRM recommended dose for the next cohort was the level with the highest posterior probability of the DLT rate falling in the target interval among the doses fulfilling escalation with overdose control criteria.

Maximum tolerated dose (MTD) was defined as the highest dose with a less than 25% risk of the true DLT rate being above 0.33. MTD was considered reached if at least one DLT was observed in the study and one of the following criteria was fulfilled: the posterior probability of the true DLT rate in that target interval of the MTD was above 0.50 or at least 15 patients had been treated in the study (of which at least six had been treated at the MTD).

DLTs observed during the first three weeks of treatment (the MTD evaluation period) of Study 1381.1 were considered for MTD determination, while the determination of the RP2D considered DLTs occurring in all treatment cycles of the study. For RP2D determination, the BLRM was re-run including the information from all cycles; the RP2D was selected after consideration of the BLRM estimates from the MTD evaluation period and the entire study period, as well as pharmacokinetic and other data obtained during the study.

Pharmacokinetic parameters were analysed by non-compartmental analysis.

## Results

### Patients and treatment

**Fig. 1:**
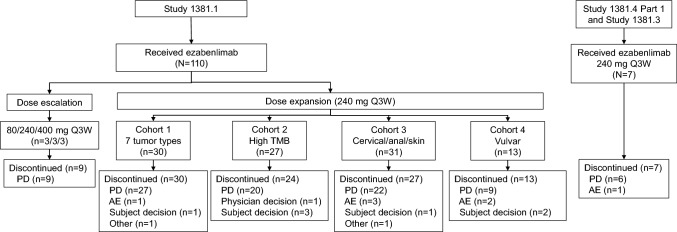
Patient disposition. AE, adverse event; PD, progressive disease; Q3W, every 3 weeks; TMB, tumour mutational burden

Between November 2016 and November 2021, 117 patients were enrolled and received ezabenlimab monotherapy. A total of 110 patients were treated in Study 1381.1 (nine in the dose escalation cohorts and 101 in dose expansion cohorts), six were treated in Study 1381.4, and one was treated in Study 1381.3 (Fig. 1). Most patients (n = 111) received ezabenlimab 240 mg Q3W, and three patients each received ezabenlimab 80 mg and 400 mg Q3W during the dose escalation part of Study 1381.1. At the data cut-off date (November 2021), 104 patients had discontinued treatment; the main reason for discontinuation was progressive disease. Seven patients remained on treatment (three patients in the high TMB cohort and four in the cervical/anal/skin cancer cohort).

The median age of the population was 61 years, the majority (73.5%) were female, and most (68.4%) had an ECOG PS of 1 (Table 1). Patients had a range of tumour types, with the most common being cervical (13.7%), ovarian (10.3%), breast (9.4%), anal (8.5%) and VSCC (7.7%). Most patients had received prior systemic therapy (95.7%), with a median of two prior regimens (range 1–10; Table 1).

**Table 1:** Patient characteristics for the treated set

Characteristic	N = 117
Gender, n (%)	
Male	31 (26.5)
Female	86 (73.5)
Median age, years (range)	61 (25–85)
Race, n (%)	
Asian	8 (6.8)
Black/African American	11 (9.4)
White	94 (80.3)
Other	4 (3.4)
Ethnicity, n (%)	
Hispanic/Latino	7 (6.0)
Not Hispanic/Latino	106 (90.6)
Unknown	4 (3.4)
ECOG PS, n (%)	
0	37 (31.6)
1	80 (68.4)
Median number of metastatic sites at screening (range)	2.5 (1–8)
Prior surgery, n (%)	72 (61.5)
Prior radiotherapy, n (%)	75 (64.1)
Prior systemic therapy, n (%)	112 (95.7)
Median number of prior systemic therapies (range)	2.0 (1–10)

ECOG PS, Eastern Cooperative Oncology Group performance status

### Maximum tolerated dose

During dose escalation in Study 1381.1, patients received ezabenlimab at three dose levels: 80 mg, 240 mg and 400 mg Q3W (n = 3 per dose level). During dose escalation, no DLTs were reported, and no MTD was reached. Per protocol, the maximum administered dose was 400 mg Q3W. Ezabenlimab 240 mg Q3W was selected as the RP2D based on an overall review of the pharmacokinetics, safety and pharmacometric modelling data from the dose escalation part of Study 1381.1.

In Part 1 of Study 1381.4, six patients received ezabenlimab 240 mg Q3W. No DLTs were reported.

### Safety and tolerability

As of the November 2021 data cut-off date, median duration of treatment for the treated patient population was 94.0 days (range 20–1008). Across the treated set (N = 117), 114 (97.4%) patients had an AE and 72 (61.5%) patients had an investigator-defined treatment-related AE (TRAE). There were no fatal AEs.

Among those who received the RP2D dose of 240 mg Q3W (n = 111), 108 (97.3%) had an AE and 67 (60.4%) had a TRAE. The most common AEs were fatigue (38.7%), nausea (29.7%) and anaemia (22.5%; Table 2). TRAEs in patients who received 240 mg Q3W are shown in Supplementary Table 1; most TRAEs were grade 1 or 2 in severity. Seven (6.3%) patients had grade 3 TRAEs and there were no grade 4 TRAEs. A total of 42 (37.8%) patients who received ezabenlimab 240 mg Q3W had serious AEs. Of these, three were considered treatment-related: grade 2 pyrexia, grade 3 drug-induced liver injury and grade 3 rash.

**Table 2:** Most common any-cause AEs with ezabenlimab 240 mg Q3W monotherapy during the on-treatment period (occurring in ≥ 10% of patients)

	All patients (N = 111)				
	All grades	Grade 1	Grade 2	Grade 3	Grade 4
Patients with any AE, n (%)	108 (97.3)	9 (8.1)	41 (36.9)	55 (49.5)	3 (2.7)^a^
Fatigue	43 (38.7)	22 (19.8)	18 (16.2)	3 (2.7)	0
Nausea	33 (29.7)	24 (21.6)	9 (8.1)	0	0
Anaemia	25 (22.5)	5 (4.5)	8 (7.2)	12 (10.8)	0
Decreased appetite	23 (20.7)	17 (15.3)	5 (4.5)	1 (0.9)	0
Diarrhoea	20 (18.0)	12 (10.8)	7 (6.3)	1 (0.9)	0
Cough	20 (18.0)	13 (11.7)	5 (4.5)	2 (1.8)	0
Constipation	20 (18.0)	13 (11.7)	7 (6.3)	0	0
Abdominal pain	18 (16.2)	6 (5.4)	10 (9.0)	2 (1.8)	0
Vomiting	17 (15.3)	14 (12.6)	3 (2.7)	0	0
Peripheral oedema	17 (15.3)	9 (8.1)	8 (7.2)	0	0
Urinary tract infection	17 (15.3)	2 (1.8)	14 (12.6)	1 (0.9)	0
Pyrexia	15 (13.5)	9 (8.1)	4 (3.6)	2 (1.8)	0
Back pain	15 (13.5)	10 (9.0)	5 (4.5)	0	0
Dyspnoea	15 (13.5)	10 (9.0)	3 (2.7)	2 (1.8)	0
Arthralgia	14 (12.6)	7 (6.3)	6 (5.4)	1 (0.9)	0
Headache	14 (12.6)	11 (9.9)	2 (1.8)	1 (0.9)	0
Rash	13 (11.7)	10 (9.0)	1 (0.9)	2 (1.8)	0
Pain in extremity	13 (11.7)	8 (7.2)	4 (3.6)	1 (0.9)	0
Hypokalaemia	13 (11.7)	11 (9.9)	0	2 (1.8)	0
Weight decreased	12 (10.8)	6 (5.4)	5 (4.5)	1 (0.9)	0
Hypothyroidism	12 (10.8)	2 (1.8)	10 (9.0)	0	0
Pruritus	12 (10.8)	10 (9.0)	2 (1.8)	0	0
Insomnia	11 (9.9)	8 (7.2)	3 (2.7)	0	0

^a^Reported G4 adverse events (preferred terms) were: hypercalcaemia; sepsis; biliary sepsis

AE, adverse event; Q3W, every 3 weeks

Immune-related AEs were reported in 31 (27.9%) patients who received ezabenlimab 240 mg Q3W during the on-treatment period (in 33 [29.7%] patients when immune-related AEs were analysed during the entire study period). The most common immune-related AEs were hypothyroidism (8 patients; 7.2%), hyperthyroidism (5 [4.5%]), maculopapular rash (5 [4.5%]) and diarrhoea [5; 4.5%]). Most of these events were grade 1 or 2 in severity. There were no grade 4 or grade 5 immune-related AEs. Five patients had grade 3 immune-related AEs (preferred terms were maculopapular rash, rash, stomatitis, aspartate aminotransferase [AST] increased, weight increased and arthralgia). No infusion-related reactions were reported. Treatment of immune-related AEs usually involved the use of corticosteroids.

Five (4.5%) patients who received ezabenlimab 240 mg Q3W had AEs leading to treatment discontinuation (grade 1 insomnia, grade 2 colitis, grade 2 AST increased, grade 2 pneumonitis and grade 3 drug-induced liver injury).

### Antitumour activity

Among the 111 evaluable patients who received ezabenlimab 240 mg Q3W, the ORR per investigator review as assessed by RECIST v1.1 was 16.2% (Table 3). Three patients had a complete response (two patients with anal cancer and one with VSCC); one of the patients with anal cancer remained free of disease and was still receiving ezabenlimab at the data cut-off date (duration of response was 906 days). Partial responses were observed in 15 patients across all Study 1381.1 cohorts (seven tumour types, high TMB, cervical/anal/skin (all n = 4), vulvar, dose escalation (all n = 1) and in Study 1381.4 (n = 1); duration of response ranged from 67 to 757 days. Stable disease was seen in 45 patients across a range of tumour types. The disease control rate was 56.8%.

**Table 3:** Best confirmed overall response in patients who received ezabenlimab monotherapy

	Study 1381.1 dose escalation			Study 1381.1 dose expansion (240 mg Q3W)				Study 1381.4 and 1381.3^a^	Total
	80 mg Q3W	240 mg Q3W	400 mg Q3W	Cohort 1 7 tumour types	Cohort 2 High TMB	Cohort 3 Cervical/anal/skin	Cohort 4 Vulvar	240 mg Q3W	
Total treated, n	3	3	3	30	27	31	13	7	117
Post-baseline assessment, n	3	3	3	29	24	31	12	6	111
Objective response, n (%)	0	1 (33.3)^b^	0	4 (13.8)^c^	4 (16.7)^d^	6 (19.4)	2 (16.7)	1 (16.7)^e^	18 (16.2)
Complete response, n (% )	0	0	0	0	0	2 (6.5)	1 (8.3)	0	3 (2.7)
Partial response, n (%)	0	1 (33.3)	0	4 (13.8)	4 (16.7)	4 (12.9)	1 (8.3)	1 (16.7)	15 (13.5)
Stable disease, n (%)	2 (66.7)	2 (66.7)	1 (33.3)	11 (37.9)	9 (37.5)	12 (38.7)	5 (41.7)	3 (50.0)	45 (40.5)
Progressive disease, n (%)	1 (33.3)	0	2 (66.7)	14 (48.3)	10 (41.7)	13 (41.9)	5 (41.7)	2 (33.3)	47 (42.3)
Not evaluable, n (%)	0	0	0	0	1 (4.2)	0	0	0	1 (0.9)
Disease control, n (%)	2 (66.7)	3 (100)	1 (33.3)	15 (51.7)	13 (54.2)	18 (58.1)	7 (58.3)	4 (66.7)	63 (56.8)

Percentages determined based on number of patients with post-baseline assessments. ^a^One patient from Study 1381.3 who received ezabenlimab included in the analysis; ^b^Tumour type: oesophageal; ^c^Tumour types: breast (n = 2), fallopian tube; renal cell carcinoma; ^d^Tumour types: angiosarcoma; breast; endometrial; left supraclavicular node; ^e^Tumour type: mesothelial

Q3W, every 3 weeks; TMB, tumour mutational burden

In the 101 evaluable patients in the dose expansion part of Study 1381.1, progression-free survival was 2.6 months.

### Pharmacokinetic profile

**Fig. 2:**
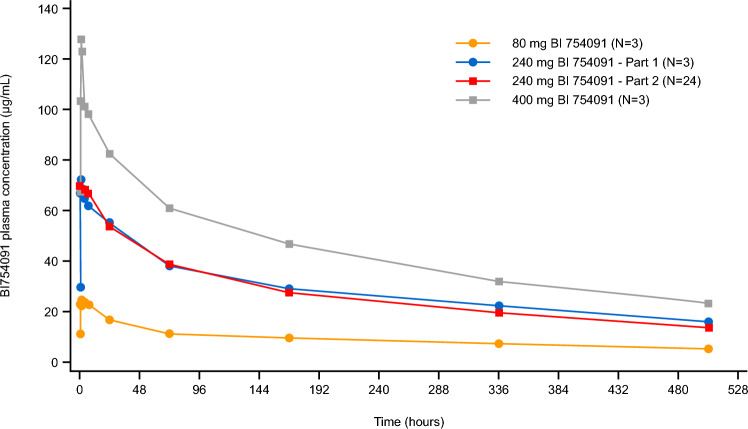
Geometric mean plasma concentration–time profiles of ezabenlimab after the first intravenous infusion during dose escalation (80, 240 and 400 mg Q3W) and dose expansion (240 mg Q3W). Q3W, every 3 weeks

The geometric mean ezabenlimab plasma concentration–time profiles of the three dose cohorts in the dose escalation part of Study 1381.1 and the 240 mg dose cohort in the expansion part are shown in Fig. 2. The profiles were at least biphasic, with a rapid distribution phase followed by a slower elimination phase. Pharmacokinetic profiles for the 240 mg dose escalation and expansion cohorts were very similar.

Pharmacokinetic parameters of ezabenlimab in Studies 1381.1 and 1381.4 are shown in Supplementary Table 2. Of note, there were no apparent differences in pharmacokinetic profiles between Japanese and Caucasian patients. ADAs occurred infrequently (in 10.2% of patients in Study 1381.1 and 0% in Study 1381.4) and did not impact the pharmacokinetic profile of ezabenlimab (data not shown).

### Pharmacodynamic assessments

Preliminary data from nine patients in the 80 mg, 240 mg and 400 mg dose groups in Study 1381.1 showed 100% PD-1 receptor occupancy in all on-treatment patient peripheral blood mononuclear cell samples versus baseline throughout the first treatment cycle. In the same treatment cycle, ezabenlimab treatment resulted in an increase in the pro-inflammatory cytokines interferon gamma (IFN-gamma), monokine induced by gamma (MIG) and interferon gamma-induced protein 10 kD (IP-10). The increases observed in the individual treatment cohorts were 1.3–7.3-fold for IFN-gamma, 1.8–4.3-fold for MIG and 1.8–3-fold for IP-10.

## Discussion

The first-in-human studies show that ezabenlimab monotherapy was well tolerated and had clinical efficacy in patients with advanced solid tumours. Ezabenlimab 240 mg Q3W was defined as the RP2D. Whilst no DLTs were observed during dose escalation (up to a dose of 400 mg Q3W), the RP2D was selected based on an overall review of the pharmacokinetics, safety and pharmacometric modelling data from the dose escalation part of Study 1381.1.

Five (4.5%) patients discontinued treatment due to AEs. At the RP2D, the most common TRAEs with ezabenlimab were fatigue and nausea; this is consistent with the known profile of other PD-1 inhibitors (where fatigue is often the most common AE and other common AEs include gastrointestinal and skin toxicities) [2, 3, 9]. Given their mechanism of action, immune-related AEs are of interest for anti-PD-1 antibodies. Based on data from approved PD-1 antibodies, these AEs most commonly include endocrinological alterations (e.g. hypothyroidism and hyperthyroidism), and gastrointestinal (e.g. colitis) and skin toxicities (e.g. rash) [10]. Consistent with this, the most common immune-related AEs in these studies were hypothyroidism, hyperthyroidism, maculopapular rash and diarrhoea. These events were manageable and generally of low-grade intensity. However, immune-related AEs may differ depending on tumour type, underscoring the importance of monitoring during the treatment period.

Although these studies were not powered for efficacy, ezabenlimab demonstrated evidence of antitumour activity in multiple tumour types. Three confirmed complete responses were observed, in one patient with VSCC and two with anal cancer. One of the patients with anal cancer remained in complete response at 906 days and was still receiving ezabenlimab at the data cut-off date. Partial responses and stable disease were observed across a range of tumour types. Durable responses were observed in several patients with various tumour types (response duration ranged from 67 to 906 days) including seven patients remaining on treatment at the data cut-off date. Taken together, the data from these studies provide an indication of the broader application of ezabenlimab that is overall consistent with other PD-1 inhibitors [11–13].

The pharmacokinetic profile was as expected for an anti-PD-1 monoclonal antibody, with ezabenlimab showing an at least biphasic distribution. The small total volume of distribution was consistent with a limited extravascular disposition as expected for a therapeutic antibody. There were also no apparent differences in the pharmacokinetic profiles between Caucasian and Japanese patients. In the pharmacodynamic analyses, no induction of any of the measured cytokines by more than 50-fold was observed; as a result, there are no cytokine-related safety concerns with ezabenlimab. Limited pharmacodynamic analyses for the paired biopsies have been performed to date. Further pharmacokinetic and pharmacodynamic data will be published separately.

The application of PD-1/PD-L1 blockade has become a milestone of cancer immunotherapy. Although anti-PD-1/PD-L1 therapy has shown impressive efficacy in the treatment of a variety of solid tumours, durable sensitivity only occurs in a small proportion of patients and some responders develop acquired resistance or relapse after initial responses. While underlying mechanisms remain largely unknown and continue to be further investigated, combinations of PD-1/PD-L1 blockade with other therapies are currently being widely evaluated with the aim to overcome the lack of response and expand the spectrum of responders to anti-PD-1/PD-L1 therapy in the future through increasing the chance of success and postponing acquired resistance [14, 15]. In line with this approach, there are several ongoing studies evaluating ezabenlimab in combination with multiple novel anticancer drug candidates with different mechanism of actions (including, but not limited to, NCT05249426 [combination with the signal-regulatory protein α antagonist, BI 765063] and NCT03964233 [combination with the murine double minute 2-tumour protein p53 antagonist, brigimadlin, BI 907828]). Outcomes from these studies are awaited and may provide further information about new combinatorial treatment approaches for cancer patients.

In summary, data from these three Phase I studies provide evidence of safety, activity, and durability of response of ezabenlimab in multiple advanced solid tumours. Ezabenlimab shows a similar safety profile and response rate to that of other PD-1 inhibitors in comparable patient populations and treatment settings. Further development of ezabenlimab in combination with a variety of anti-cancer drug candidates is being pursued in multiple tumour types.

### Supplementary Information

Below is the link to the electronic supplementary material.

Supplementary file1 (PDF 145 KB)

## Data Availability

To ensure independent interpretation of clinical study results and enable authors to fulfil their role and obligations under the ICMJE criteria, Boehringer Ingelheim grants all external authors access to relevant clinical study data. In adherence with the Boehringer Ingelheim Policy on Transparency and Publication of Clinical Study Data, scientific and medical researchers can request access to clinical study data, typically, one year after the approval has been granted by major Regulatory Authorities or after termination of the development program. Researchers should use the https://vivli.org/ link to request access to study data and visit https://www.mystudywindow.com/msw/datasharing for further information.
