# Supplementary material for: Ezabenlimab (BI 754091), an anti-PD-1 antibody, in patients with advanced solid tumours
Source: Cancer Immunol Immunother. 2024 Mar 30;73(5):89. doi: 10.1007/s00262-024-03654-0 (PMC10981579; doi:10.1007/s00262-024-03654-0)
Supplement: Supplementary file 1 — Supplementary file1 (PDF 145 KB) [file 262_2024_3654_MOESM1_ESM.pdf]

**Supplementary Materials**

**Cancer Immunology, Immunotherapy (submitted in March 2023) – Manish R. Patel, et al.**

**Supplementary Table 1.** Most common treatment-related adverse events with ezabenlimab 240 mg Q3W monotherapy during the on-treatment period (occurring in ≥5% of patients).

| | All patients (N=111)[a] | | | |
|---|---|---|---|---|
| | **All grades** | **Grade 1** | **Grade 2** | **Grade 3** |
| Patients with any TRAE, n (%) | 67 (60.4) | 28 (25.2) | 32 (28.8) | 7 (6.3) |
| Fatigue | 21 (18.9) | 13 (11.7) | 8 (7.2) | 0 |
| Nausea | 11 (9.9) | 9 (8.1) | 2 (1.8) | 0 |
| Diarrhoea | 9 (8.1) | 4 (3.6) | 4 (3.6) | 1 (0.9) |
| Hypothyroidism | 9 (8.1) | 2 (1.8) | 7 (6.3) | 0 |
| Myalgia | 7 (6.3) | 5 (4.5) | 2 (1.8) | 0 |
| Rash | 7 (6.3) | 5 (4.5) | 0 | 2 (1.8) |
| Hyperthyroidism | 6 (5.4) | 4 (3.6) | 2 (1.8) | 0 |
| Pruritus | 6 (5.4) | 4 (3.6) | 2 (1.8) | 0 |

[a]There were no grade 4 or 5 TRAEs.

Q3W, every 3 weeks; TRAE, treatment-related adverse event.

**Supplementary Table 2.** Pharmacokinetic parameters for ezabenlimab in Studies 1381.1 and 1381.4.

| Parameter<br>Geometric mean (% gCV) | n | 1381.1<br>80 mg | n | 1381.1<br>240 mg, part 1 | n | 1381.1<br>240 mg, part 2[a] | n | 1381.1<br>400 mg | n | 1381.4<br>240 mg, part 1<br>Asian |
|---|---|---|---|---|---|---|---|---|---|---|
| **Cycle 1** | | | | | | | | | | |
| $AUC_{0\text{-}504}$ [µg*h/mL] | 3 | 4640 (28.0) | 3 | 14,300 (19.6) | 22 | 13,600 (22.2) | 3 | 22,000 (31.9) | 6 | 15,400 (46.6) |
| $AUC_{0\text{-}504,norm}$ [µg*h/mL/mg] | 3 | 58 (28.0) | 3 | 59.7 (19.6) | 22 | 56.7 (22.2) | 3 | 55 (31.9) | 6 | 64.2 (46.6) |
| $C_{max}$ [µg/mL] | 3 | 25.3 (32.7) | 3 | 73.7 (9.93) | 24 | 73.1 (19.5) | 3 | 128 (27.1) | 6 | 78.6 (33.7) |
| $C_{max,\ norm}$ [µg/mL/mg] | 3 | 0.316 (32.7) | 3 | 0.307 (9.93) | 24 | 0.305 (19.5) | 3 | 0.321 (27.1) | 6 | 0.328 (33.7) |
| $C_{trough}$ (=$C_{504}$) [µg/mL] | 3 | 5.420 (22.5) | 3 | 15.9 (26.5) | 22 | 13.1 (50.8) | 3 | 23.1 (49.6) | 6 | 16.1 (68.7) |
| $C_{trough,\ norm}$ [µg/mL/mg] | 3 | 0.0678 (22.5) | 3 | 0.0663 (26.5) | 22 | 0.0546 (50.8) | 3 | 0.0578 (49.6) | 6 | 0.0671 (68.7) |
| $t_{max}$ [h][b] | 3 | 1.52 (1.50–4.00) | 3 | 1.50 (1.33–2.00) | 24 | 2.53 (0.967–7.03) | 3 | 1.52 (1.50–2.00) | 6 | 1.25 (1.00–2.00) |
| CL [L/h] | 3 | 0.00987 (26.6) | 3 | 0.0102 (22.4) | 24 | 0.0116 (37.0) | 3 | 0.0119 (45.7) | 6 | 0.0101 (63.2) |
| $V_{ss}$ [L] | 3 | 5.88 (30.4) | 3 | 5.51 (23.5) | 24 | 5.29 (18.9) | 3 | 5.56 (20.1) | 6 | 4.71 (35.3) |
| $t_{½}$ [days] | 3 | 17.8 (17.0) | 3 | 16.3 (25.1) | 24 | 13.9 (38.0) | 3 | 14.1 (21.6) | 6 | 13.8 (32.3) |
| **Cycle 2** | | | | | | | | | | |
| $AUC_{0\text{-}504}$ [µg*h/mL] | 3 | 6750 (19.0) | 2[c] | 18,800 (21.5) | 16 | 19,400 (29.6) | 3 | 28,500 (37.1) | 6 | 21,400 (50.0) |
| $AUC_{0\text{-}504,norm}$ [µg*h/mL/mg] | 3 | 84.4 (19.0) | 2[c] | 78.1 (21.5) | 16 | 81 (29.6) | 3 | 71.2 (37.1) | 6 | 89 (50.0) |
| $C_{max}$ [µg/mL] | 3 | 33.3 (23.7) | 3 | 96.4 (18.3) | 23 | 88.1 (25.6) | 3 | 144 (21.9) | 6 | 105 (28.7) |
| $C_{max,\ norm}$ [µg/mL/mg] | 3 | 0.416 (23.7) | 3 | 0.402 (18.3) | 23 | 0.367 (25.6) | 3 | 0.361 (21.9) | 6 | 0.437 (28.7) |
| $C_{trough}$ (=$C_{504}$) [µg/mL] | 2[c] | 7.470 (52.6) | 2[c] | 21.8 (32.4) | 15 | 21.4 (52.0) | 3 | 28.4 (86.5) | 6 | 25.5 (74.4) |
| $C_{trough,\ norm}$ [µg/mL/mg] | 2[c] | 0.0934 (52.6) | 2[c] | 0.0908 (32.4) | 15 | 0.0892 (52.0) | 3 | 0.0710 (86.5) | 6 | 0.106 (74.4) |
| $t_{max}$ [h][b] | 3 | 1.47 (0.917–1.67) | 3 | 1.00 (0.983–1.48) | 23 | 4.00 (0.950–7.13) | 3 | 1.52 (1.05–2.00) | 6 | 1.50 (1.00–7.00) |

n, number of observations considered for the descriptive statistics. [a]Part 2 includes the first 24 patients of Cohort 4; [b]Median (min-max), planned time for 1381.4; [c]gMean calculated based on n=2.

$AUC_{0\text{-}504}$, area under the plasma concentration–time curve from time 0 to 504 hours; $AUC_{0\text{-}504,norm}$, normalised area under the plasma concentration–time curve from time 0 to 504 hours; CL, clearance; $C_{max}$, maximum plasma concentration; $C_{max,norm}$, normalised maximum plasma concentration; $C_{trough}$, trough plasma concentration; $C_{trough,norm}$, normalised trough plasma concentration; gCV, geometric coefficient of variation; $t_{½}$, terminal half life; $t_{max}$, time of maximum plasma concentration; $V_{ss}$, volume of distribution at steady state.
